# Bisphosphonates for osteoporosis treatment are associated with reduced breast cancer risk

**DOI:** 10.1038/sj.bjc.6605555

**Published:** 2010-02-16

**Authors:** P A Newcomb, A Trentham-Dietz, J M Hampton

**Affiliations:** 1 University of Wisconsin Paul P Carbone Comprehensive Cancer Center, 610 Walnut Street, Madison, WI 53726, USA; 2 Cancer Prevention Program, Fred Hutchinson Cancer Research Center, 1100 Fairview Ave N, M4-B402, PO Box 19024, Seattle, WA 98109, USA

**Keywords:** bisphosphonates, osteoporosis, breast cancer, case–control

## Abstract

**Background:**

Bisphosphanates are used primarily for the prevention and treatment of osteoporosis, and are also indicated for osseous complications of malignancy. In addition to their bone resorption properties, the most commonly used nitrogen-containing bisphosphonate compounds also inhibit protein prenylation, and thus may exert anti-tumour properties.

**Methods:**

To evaluate whether the use of these drugs may be associated with cancer, specifically breast cancer, we conducted a population-based case–control study in Wisconsin from 2003 to 2006. Participants included 2936 incident invasive breast cancer cases and 2975 population controls aged <70 years. Bisphosphonate use and potential confounders were assessed by interview.

**Results:**

Using multivariable logistic regression, the odds ratio for breast cancer in current bisphosphonate users compared with non-users was 0.67 (95% confidence interval 0.51–0.89). Increasing duration of use was associated with a greater reduction in risk (*P*-trend=0.01). Risk reduction was observed in women who were not obese (*P*-interaction=0.005).

**Conclusion:**

These results are suggestive of an additional benefit of the common use of bisphosphonates, in this instance, the reduction in breast cancer risk.

Bisphosphonates are commonly prescribed for the prevention and treatment of osteoporosis (Rosen, 2005). By inhibiting bone resorption, bisphosphonate therapy also reduces skeletal complications from bone involvement in breast, prostate, and other cancers, and from the bone-depleting consequences of some treatments (Berenson *et al*, 1996; Aapro *et al*, 2008; Saad *et al*, 2008). In addition to the reduction in osteoclastic bone resorption, the more potent aminobisphosphonates inhibit the mevalonate pathway, thereby affecting cell function and survival(Luckman *et al*, 1998; Dunford *et al*, 2001). As this pathway leads to the prenylation of key signalling proteins required by all cells, aminobisphosphonates may affect the viability of other cell types, such as tumours (Russell *et al*, 1999; Caraglia *et al*, 2006). Nitrogen-containing bisphosphonates have also been shown to directly induce tumour apoptosis, inhibit angiogenesis, and prevent tumour cell adhesion (Neville-Webbe *et al*, 2002; Wood *et al*, 2002). These anti-proliferative actions may be relevant to the development of a broad range of cancers, including breast neoplasms(Green, 2003; Winter *et al*, 2008). We examined the association between bisphosphonate use and breast cancer in a population-based case–control study.

## Materials and methods

Women aged 20–69 years with a new diagnosis of invasive breast cancer from 2003 to 2006 were identified from Wisconsin's mandatory cancer registry (Sprague *et al*, 2008; Wernli *et al*, 2009). For comparison, similarly aged community women without a personal history of breast cancer were randomly selected from driver's licence lists. The study included 2988 breast cancer cases (74% of eligible) and 3004 controls (67% of eligible). Women were invited to participate in a structured interview to ascertain the use of bisphosphonates, type of drug, duration and recency of use, as well as physician diagnosis of osteoporosis, history of fractures, height change since the age of 18 years, and other risk factors for breast cancer. Exposure information was limited to events before a reference date that was defined as the date of diagnosis for cases and, for comparability, the date approximately 1 year before interview for control women. The study was approved by the University of Wisconsin-Madison institutional review board.

Multivariable logistic regression models were used to estimate odds ratios and 95% confidence intervals and tests for linear trend across ordinal values of categorical variables. Current use of bisphosphonates was defined as use in the year before the reference year, and former users had taken bisphosphonates at any time before this period. Duration of use was the sum of all periods of bisphosphonate use in the year before the reference period. Analyses were adjusted for potentially confounding variables selected *a priori*: age at reference date, year of interview, parity (0–1, 2, 3, 4+), age at first birth (nulliparous, <20, 20–24, 25–29, 30+), first-degree family history of breast cancer, body mass index 1 year before reference date (<24.9, 25–29.9, ⩾30 kg m^–2^), menopausal status, age at menopause (<45, 45–49, 50–54, 55+), screening mammograms over the past 5 years (0, 1–4, ⩾5), a physician diagnosis of osteoporosis, smoking status, and height change from the age of 18 years. Effect modification was evaluated by inclusion of cross-product interaction terms in logistic models. Owing to missing or incomplete information on osteoporosis medications or their indications, 26 breast cancer cases and 29 control women were excluded, leaving 2936 cases and 2975 controls for analysis.

## Results

The average age at the reference date for breast cancer cases was 54.2 years (s.d.=8.9 years) and for controls it was 54.8 years (8.9 years). As expected, women with breast cancer were more likely than controls to have a family history of breast cancer, a college degree, to be older at first birth or nulliparous, to have gained more weight since the age of 18 years, and to use postmenopausal hormones; controls were more likely to report a physician's diagnosis of osteoporosis (data not shown).

Bisphosphonates were more commonly used by controls than by cases; the multivariate-adjusted odds ratio for ever use was 0.70 (95% confidence interval 0.54–0.92; Table 1), which was similar to the age-adjusted odds ratio, suggesting that the influence of confounding was limited. The reduction in risk was significant only among current users of bisphosphonates, although few women were former users at the reference date. Increasing duration of use was associated with greater reductions in breast cancer risk (*P-*trend=0.01).

**Figure 1:**
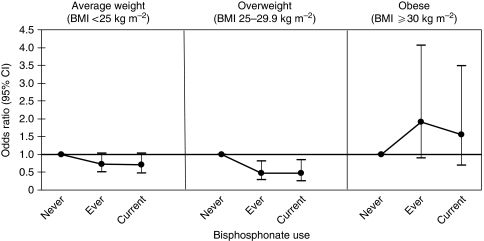
Odds ratios and 95% confidence intervals of breast cancer according to bisphosphonate use and body mass index (BMI). Odds ratios were adjusted for age at diagnosis, party, age at first live birth, family history of breast cancer, BMI menopausal status, age at menopause, type of hormone use, mammography, osteoporosis, smoking history, and height change. *P*-interaction for BMI and use of bisphosphonates=0.005.

To evaluate whether the inverse effects of bisphosphonates were attributable to the medications themselves or to the conditions for which they were prescribed, we examined use according to fracture history (excluding women diagnosed with distant disease), height loss, and physician-diagnosed osteoporosis. There was a suggestion that use of bisphosphonates was associated with a reduced risk of breast cancer only among women reporting symptoms of bone loss (postmenopausal fractures, osteoporosis, and height loss), but the differences were not statistically significant (*P-*interaction=0.29). The inverse effect of bisphosphonates seemed limited to women who were not obese (*P*-interaction=0.005, Figure 1). There were no statistical interactions between bisphosphonates and age, smoking, or hormone therapy (data not shown).

## Conclusion

In this case–control study, use of bisphosphonates was associated with approximately a 30% reduction in breast cancer risk, and this decrease in risk was greatest for longer durations of use, and among leaner women. This association was not attributed to the primary indication for use, bone density loss, or fractures, which we and others have previously shown as a risk factor for breast cancer (Cauley *et al*, 1996; Newcomb *et al*, 2001; Silverman *et al*, 2004). Our confidence in these results is strengthened by the large size of the study, the population-based sampling, our consideration of important confounders such as body mass index and postmenopausal hormone use, and the generally high reliability and validity of self-reports of medication use (Lipworth *et al*, 2001; Boudreau *et al*, 2004, 2005).

There are limited data that directly support our epidemiological findings. However, a number of observations may be relevant to a protective effect of these medications on breast cancer incidence. In animals bearing breast cancer xenografts, bisphosphonates block the growth of bone metastases, and also extraskeletal disease (Senaratne *et al*, 2000; Hiraga *et al*, 2001). Indeed, direct cytotoxicity seen *in vitro* (Fromigue *et al*, 2000) may account for some of the clinical benefit in patients taking bisphosphonates, specifically aminobisphosphonates (Neville-Webbe *et al*, 2002; Green, 2003; Caraglia *et al*, 2004). Although the molecular targets of bisphosphonates are not fully known, several phases of tumour growth and progression seem to be influenced (Neville-Webbe *et al*, 2002; Wood *et al*, 2002; Caraglia *et al*, 2006). In a recent clinical trial of early-stage breast cancer, nitrogen-containing bisphosphonates seemed to improve long-term outcomes (Gnant *et al*, 2009), and several large clinical trials are currently underway to clarify this issue (Bedard *et al*, 2009). Our finding that bisphosphonates were not associated with breast cancer risk reductions in obese women suggests that their inhibitory actions may be related to some threshold effect of hormonal or other growth factors; these are important aetiological exposures in breast cancer (Hankinson *et al*, 2004). Thus, the pluripotent actions of bisphosphonates are consistent with our observed findings.

Unmeasured or unknown confounding factors may have been present, for which adjustment was not possible. Although we inquired about clinical symptoms and reports of physician-diagnosed osteoporosis, bone mineral density was not available. Nearly all users had used aminobisphosphonates (97% of users); therefore, we could not evaluate specific types of preparations that have very different mechanisms of action (Russell and Rogers, 1999; Bedard *et al*, 2009). Sample size was also too modest to evaluate other breast cancer subgroups that may experience different responses to bisphosphonates, such as specific histological type or hormone receptor status. Finally, because the study population was limited to women under the age of 70 years, the prevalence of bisphosphonate use was lower than might be expected in older women with clinical osteoporosis.

In summary, this study provides new evidence that the use of bisphosphonates is associated with a potentially important reduction in breast cancer risk. Because we were able to account for important confounders, these findings may reflect real benefits due to the anti-tumour mechanisms of these medications.

## Figures and Tables

**Table 1:** Odds ratios (ORs) (95% confidence intervals, CIs) of breast cancer associated with bisphosphonate use

	**Cases (*N*=2936)**	**Controls (*N*=2975)**	**Age adjusted**	**Multivariable adjusted**
***Bisphosphonate use***	***N* (%)**	***N* (%)**	**OR (95% CI)^a^**	**OR (95% CI)^b^**
Never use	2808 (95.6)	2788 (93.7)	1.00 (Referent)	1.00 (Referent)
*Ever use*	128 (4.4)	187 (6.2)	0.70 (0.56–0.89)	0.70 (0.54–0.92)
Former	22 (0.7)	26 (0.9)	0.85 (0.48–1.51)	0.91 (0.50–1.64)
Current	106 (3.6)	161 (5.4)	0.68 (0.53–0.88)	0.67 (0.51–0.89)
				
*Duration of use (months)*				
3–12	58 (2.0)	76 (2.5)	0.78 (0.55–1.11)	0.78 (0.54–1.12)
13–24	26 (0.9)	37 (1.2)	0.72 (0.44–1.20)	0.69 (0.41–1.17)
⩾25	44 (1.5)	74 (2.5)	0.61 (0.42–0.90)	0.63 (0.42–0.95)
*P*-trend			0.01	0.01

a Adjusted for age at diagnosis.

b Additionally adjusted for parity, age at first live birth, family history of breast cancer, body mass index, menopausal status, age at menopause, type of hormone use, mammography, osteoporosis, smoking history, and height change.
